# The heterogeneous multiscale method applied to inelastic polymer mechanics

**DOI:** 10.1098/rsta.2018.0150

**Published:** 2019-02-18

**Authors:** M. Vassaux, R. A. Richardson, P. V. Coveney

**Affiliations:** Centre for Computational Science, Department of Chemistry, University College London, 20 Gordon St, WC1H 0AJ London, UK

**Keywords:** heterogeneous multiscale method, polymer mechanics, fracture, inelasticity, high performance computing

## Abstract

Mechanisms emerging across multiple scales are ubiquitous in physics and methods designed to investigate them are becoming essential. The heterogeneous multiscale method (HMM) is one of these, concurrently simulating the different scales while keeping them separate. Owing to the significant computational expense, developments of HMM remain mostly theoretical and applications to physical problems are scarce. However, HMM is highly scalable and is well suited for high performance computing. With the wide availability of multi-petaflop infrastructures, HMM applications are becoming practical. Rare applications to mechanics of materials at low loading amplitudes exist, but are generally confined to the elastic regime. Beyond that, where history-dependent, irreversible or nonlinear mechanisms occur, not only computational cost but also data management issues arise. The micro-scale description loses generality, developing a specific microstructure based on the deformation history, which implies *inter alia* that as many microscopic models as discrete locations in the macroscopic description must be simulated and stored. Here, we present a detailed description of the application of HMM to inelastic mechanics of materials, with emphasis on the efficiency and accuracy of the scale-bridging methodology. The method is well suited to the estimation of macroscopic properties of polymers (and derived nanocomposites) starting from knowledge of their atomistic chemical structure. Through application of the resulting workflow to polymer fracture mechanics, we demonstrate deviation in the predicted fracture toughness relative to a single-scale molecular dynamics approach, thus illustrating the need for such concurrent multiscale methods in the predictive estimation of macroscopic properties.

This article is part of the theme issue ‘Multiscale modelling, simulation and computing: from the desktop to the exascale’.

## 1. Introduction

The design and manufacture of advanced multifunctional materials remains a slow, uncertain, expensive and time-consuming process. The current approach, largely unchanged in decades, consists of an extensive trial-and-error process followed by testing and certification. This process typically takes around 20 years from concept to commercial implementation [1,2]. Computational models offer, in principle, a serious alternative due to their ability to perform parametric studies with high efficiency [3,4]. Yet, applicable models still suffer from major drawbacks in terms of accuracy, chemical specificity and reliability [5,6]. Effective integration within the design process requires computational models to be certified via appropriate validation and verification, and the results rendered actionable via uncertainty quantification.

The macroscopic mechanics of solids are highly dependent on structural detail found at each scale, from the electronic organization at the quantum mechanical level through to the geometry of the sample at the macroscopic scale. Attempts to estimate macroscopic mechanical properties using a single model at nanoscopic resolution are quickly rendered intractable by the astronomical computational cost or limited by unrealistic test set-ups involving, for example, microscopic material samples or simplistic uniaxial tests. Scale-bridging approaches become essential [7,8].

To render such simulations tractable, so-called hierarchical multiscale models make use of a loosely coupled scheme in which continuum-level constitutive equations are parametrized beforehand using lower-level molecular or coarse-grained simulations [9–12]. However, in trying to reproduce the wide variety of lower-scale mechanisms occurring, these empirically formulated constitutive equations suffer vast increases in complexity, becoming ill-defined and difficult to integrate [5,13]. As a consequence, the constitutive equations are reduced to a massively simplified description of the true behaviour of the material, potentially missing out important phenomena that appear under non-trivial mechanical states at the microscopic level.

As a (partial) remedy to these problems, computational schemes have been developed that seamlessly couple multiple scales in a semi-concurrent fashion, implemented through the heterogeneous multiscale method (HMM) [14–16]. A similar scheme built on two finite-element models is the finite-element squared (FE2) method [17,18]. The scheme has been classified as semi-concurrent [19] because the multiple models are run in parallel but solved separately, in contradistinction to concurrent methods which embed a discrete and a continuum description within a single system [20,21]. HMM and other semi-concurrent methodologies have the benefit of being extremely scalable, as the lower-scale models are independent of each other and can therefore be simulated in parallel. For example, one may perform thousands of molecular dynamics (MD) simulations (representing the lower-scale system) simultaneously coordinated by an upper-scale model.

Owing to the increases in the computational power and parallelism available on the biggest supercomputers, as well as sophisticated computational workflows [22,23], such computational methods that were unrealistic only a few years ago may now be used to assess the mechanical properties of solids in an engineering context, while retaining atomistic-level chemical specificity. Nevertheless, implementations of HMM bridging such scales [24–27] are rare and have, to date, been limited to elastic mechanics. We propose here modifications to the standard HMM implementation to capture history-dependent mechanical behaviour, including nonlinear and irreversible mechanisms. In an attempt to analyse or predict mechanical properties related to failure, fatigue, energy dissipation (and many other situations), capturing these inelastic mechanisms is of the utmost importance. We present these enhancements to the HMM workflow with emphasis on data transfer between scales and data management of single-scale models. We then demonstrate the benefits of such an approach to the quantification of macroscopic mechanical properties, such as toughness, as opposed to those derived from single-scale atomistic approaches (e.g. [28–31]).

The developments presented here are applied to the two-scale modelling of thermoset polymers, relying on a MD [32] description of the material at the lower, atomistic scale and a finite-element method (FEM) [33,34] description at the upper, continuum scale. We explore in detail the consequences of solving inelastic mechanics on the simulation of the atomistic model.

## 2. Single-scale models

**Figure 1.**
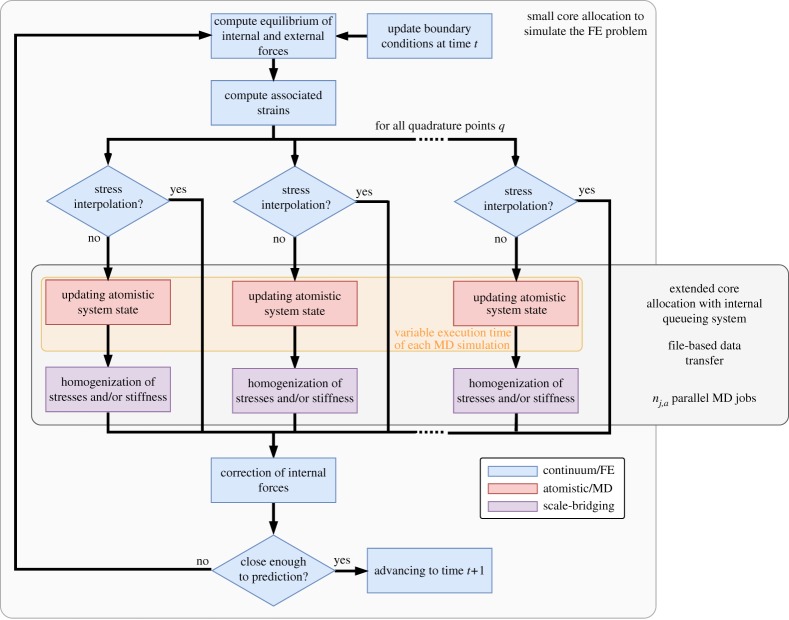
Global workflow of the multiscale model based on HMM. The building blocks of the workflow include: a continuum (FEM) model (in blue), an atomistic (MD) model (in red) and a scale-bridging coupling procedure based on homogenization (in violet); the cyclic coupling procedure involves a dynamic number of atomistic model simulations *n*^*t*^_md_ at each time-step *t*. (Online version in colour.)

The two single-scale models are orchestrated in a cyclic dynamic fashion (figure 1). Each iteration (time-step) of the continuum model requires the parallel execution of a large number of atomistic-level models *n*^*t*^_md_ with variable execution time.

The atomistic models are simulated using all-atom MD [35], a method that integrates Newton's equations of motion (2.1) for a system of individually resolved atoms:

2.1 $$-\nabla U\left(r_{i}^{a}\right)=m_{i}^{a}\frac{d^{2}r_{i}^{a}}{dt^{2}}$$

with ***r***^*a*^_*i*_ the displacements of the atom *i*, *U* the potential energy of the thermodynamic ensemble, *m*^*a*^_*i*_ the mass of the atom *i* of the atomic model.

In the present case, for temperature control, stochastic equations of Langevin dynamics (2.2) are solved:

2.2 $$-\nabla U\left(r_{i}^{a}\right)-\gamma \frac{dr_{i}^{a}}{dt}+\sqrt{2\gamma k_{B}T}R\left(t\right)=m_{i}^{a}\frac{d^{2}r_{i}^{a}}{dt^{2}}$$

with *γ* a damping parameter, *T* the temperature of the thermodynamic ensemble, *k*_B_ the Boltzmann constant and ***R***(*t*) a stationary Gaussian process with a zero time average. The degrees of freedom of the atomistic system ***r***^*a*^_*i*_ are the displacements of the particles representing the atoms, subject to constraints imposed by the molecular structure.

We simulate the system as a canonical ensemble (NVT), which conserves the number of atoms (*N*), and fixes the volume (*V* ) and temperature (*T*) of the system. The atomistic model is initialized in its most recent configuration of atoms. As we deal with inelastic mechanics, the system cannot be started from an equilibrium configuration, as the history must be preserved. Updating the atomistic mechanical state is performed by deforming the system box according to the strain tensor ***ϵ*** provided by the continuum model. The volume is varied by applying a constant strain rate ${\dot{\epsilon }}_{a}$ on the system, which is chosen as a function of the characteristic relaxation time *τ*_*a*_ of the material (this will be the object of further discussion in the following sections). As a result, the number of time-steps to perform in each atomistic model simulation will, in general, be directly proportional to the amplitude of the applied deformation (the length of time-step being constant). Coarse-grained MD is not considered in the present study, as the process of coarse-graining the atomistic system requires recent parametrization techniques to capture mechanisms of interest such as fracture [36]. However, all-atom MD models could easily be replaced by coarse- grained ones.

The continuum system is discretized using a generally unstructured mesh on which the FEM is used to solve the linear momentum conservation equation for a locally homogeneous solid (2.3):

2.3 $$\nabla \sigma \left(x\right)+\rho f\left(x\right)=\rho \frac{d^{2}x}{dt^{2}}$$

with *ρ* the density of the continuum and ***f*** the volumetric forces.

Boundary conditions are applied on the position of nodes of the mesh of the model ***x***, namely the nodal displacements or on the nodal forces. The system is parametrized by the local strains (represented by the second-order tensors) or the local stresses. Classically, the strains and stresses are linked by means of constitutive equations, but in the present case that relation is established via the response of atomistic models.

## 3. Scale-bridging for inelastic mechanics

The cornerstone of the proposed work relies on a scale-bridging strategy that benefits from the separation of time and spatial scales. Moreover, the proposed strategy includes efficient management of the data exchanged between the single-scale models and a tunable sampling methodology for the atomistic simulations.

### (a) Separation of scales

Scale separation is a fundamental criterion for the applicability of HMM, allowing the mechanics of the atomistic scale (at a given time and location within the continuum model) to be analysed at greatly reduced cost [37].

**Figure 2.**
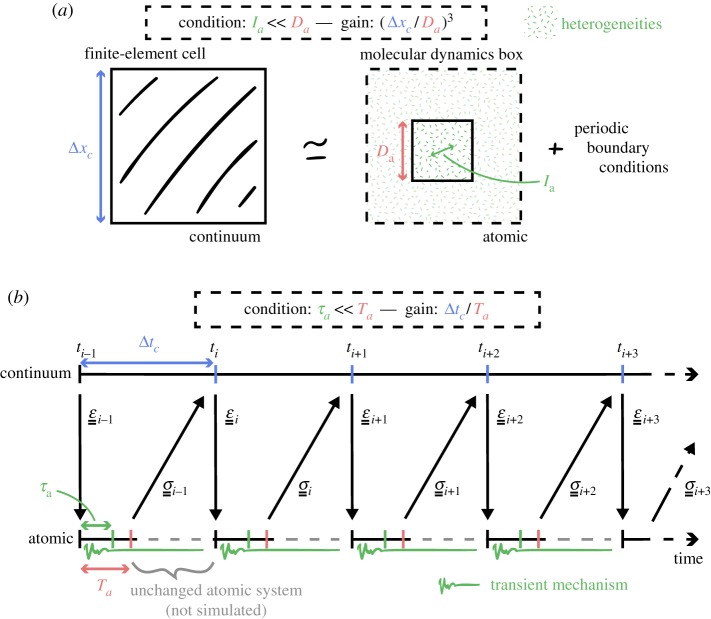
(*a*) Periodic boundary conditions applied to an atomistic system encompassing a few dimensions of the longest heterogeneity *l*_*a*_ avoid the simulation of the atomistic model on the whole volume of a cell of the mesh of the continuum system; (*b*) asynchronous coupling of atomistic and continuum time scales shortens the evolution of the atomistic system to durations of the order of the atomistic relaxation time *τ*_*a*_. (*a*) Spatial scale-bridging, (*b*) time scale-bridging.

**Figure 3.**
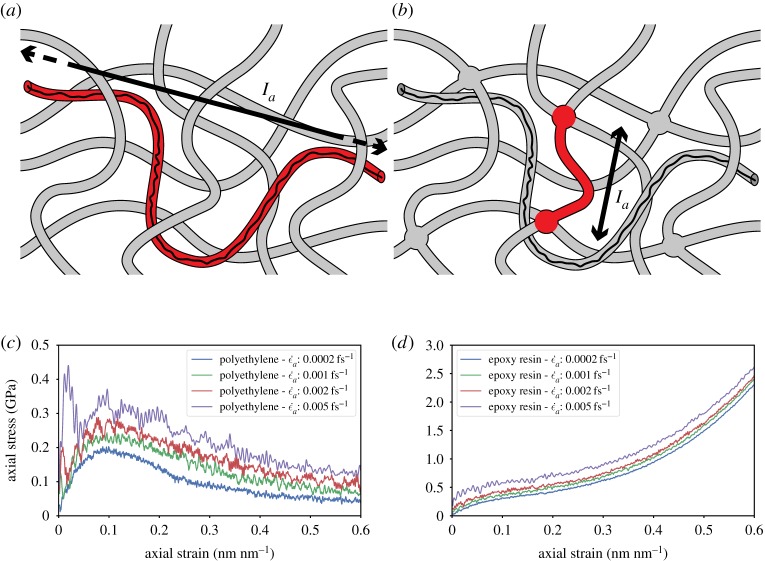
Comparison of the structure of (*a*) a thermoplastic and (*b*) a thermoset polymer; for a given polymer chain, the process of cross-linking reduces the atomistic characteristic length *l*_*a*_ of the system from the entanglement length to the distance between two cross-links, and largely inhibits the sliding of chains past each other (by a reptation mechanism) therefore reducing relaxation time (based on the illustration by Henderson C., 2015, CC0). The constitutive behaviour of (*c*) short-chain polyethylene, a thermoplastic polymer, and (*d*) TGMDA epoxy, a thermoset polymer, for different strain rates ${\dot{\epsilon }}_{a}$; decreasing the strain rate ${\dot{\epsilon }}_{a}$ ceases to have an influence comparatively earlier for epoxy resin than for polyethylene, convergence of the behaviour appearing to be hardly reached for polyethylene in the range of test strain rates. (*a*) Polyethylene reptation length, (*b*) epoxy cross-link distance, (*c*) polyethylene uniaxial behaviour and (*d*) epoxy uniaxial behaviour.

In spatial terms, it is not necessary to compute over the entire volume of the associated finite-element cell, but rather over a greatly reduced subset of that, corresponding to the scale over which the system can be treated as effectively homogeneous. Equivalently, these are the dimensions for which the volume simulated is far greater than the largest heterogeneous feature at that scale. Boundary conditions are commonly applied to this reduced system, enforcing this assumption of infinite replicability. In polymer systems, the dimension of the largest heterogeneity relates to the entanglement length in the case of thermoplastics, and to the distance between cross-links in the case of thermosets. The required dimensions for a simulation of thermosets are therefore far smaller in general than those required in the case of thermoplastic polymers. An indirect consequence of the spatial scale separation is that the volume of the continuum system is simulated by many independent atomistic models. These simulations are coordinated at each iteration of the continuum model. As a result, the overall simulation workflow is highly scalable, as all the atomistic model simulations required at a given time-step can be performed in parallel and independently (figures 2 and 3).

Similarly, with regard to separation of time scales, the atomistic dynamics does not need to be simulated for the length of an entire time-step of the macroscale (continuum) model, but rather only until the system relaxes and the stress tensor becomes stationary on average . As a direct consequence of considering inelastic mechanics, the time *τ*_*a*_ to reach a relaxed state may become large, especially for viscoelastic materials such as polymers, even at small scales. Once again, the type of polymer will influence greatly the time to reach convergence. The introduction of cross-links greatly impedes long, diffusive mechanisms such as reptation, thereby accelerating the relaxation. The strain rate is usually the prevailing factor in the case of polymer mechanics. ${\dot{\epsilon }}_{c}$ is necessarily not preserved through the atomistic models, but convergence of the constitutive behaviour (that is the stress–strain relationship) to the quasi-static case can be found at relatively high ${\dot{\epsilon }}_{a}$.

The equations (3.1) illustrate the constraints on the time *T*_*a*_ and spatial *D*_*a*_ dimensions over which to simulate the atomistic system, depending on the relaxation time *τ*_*a*_ and size of heterogeneities *l*_*a*_ at the atomistic scale:

3.1 $$\begin{array}{r} l_{a}\ll D_{a},\;\tau_{a}\ll T_{a}\\ \;\mathrm{and}\;\;g_{x}=\frac{\Delta x_{c}}{D_{a}},\;g_{t}=\frac{\Delta t_{c}}{T_{a}} \end{array}\}$$

They also define the computational reduction in time *g*_*t*_ and space *g*_*x*_ with respect to the atomistic dimensions *T*_*a*_ and *D*_*a*_, and the continuum scale time Δ*t*_*c*_ and spatial Δ*x*_*c*_ increments.

It is through the concept of separation of scales that the HMM scheme achieves a major reduction in computational cost. With regard to simulating the whole volume of the continuum system at an atomic resolution (both in time and in space), the computations are reduced twofold. First, the reduction in time is on the order of the ratio of the relaxation time of the atomistic system to the time-step of the continuum model. And second, the reduction in space is on the order of the ratio of the characteristic length of the heterogeneities in the atomistic system to the spatial discretization length of the continuum model. The only overhead incurred by the HMM is then the simulation of the continuum model over an overlapping spatio-temporal domain. But the cost of the simulation of the continuum model is negligible compared to the cost of the many atomistic models; therefore, this is never a limitation of HMM.

### (b) Data management

In this two-level scheme, the upper and lower scales evolve separately but must exchange information on a regular basis. Any given point in the continuum model has its own, local mechanical history, necessitating (in general) a unique instance (or set of instances) of the atomistic model representing the molecular-level mechanics associated with that region. This prevents the reconstruction of the atomistic system in the current mechanical state every time an atomistic model needs to be simulated [38,39]. In the finite-element discretization of this continuum, these points correspond to the quadrature points *q* in each finite element. The local mechanical states may exhibit irreversible and nonlinear behaviour (such as cracking or plastic deformation) necessitating the local state to persist between successive simulations at a given location. This means the most recent configuration of atoms must be stored for each *q*, so that it may be used as the starting point for any future atomistic simulations required during evolution of the continuum model. As the continuum model algorithm is incremental in time, we avoid the need to store the complete configurational history of each atomistic model, instead only retaining the mechanical state at the end of the most recent step.

The combined effect of this is that the atomistic models simulate essentially continuous trajectories, but under varying boundary conditions determined by solving the balance of forces at the continuum level.

### (c) Data transfer across scales

**Figure 4.**
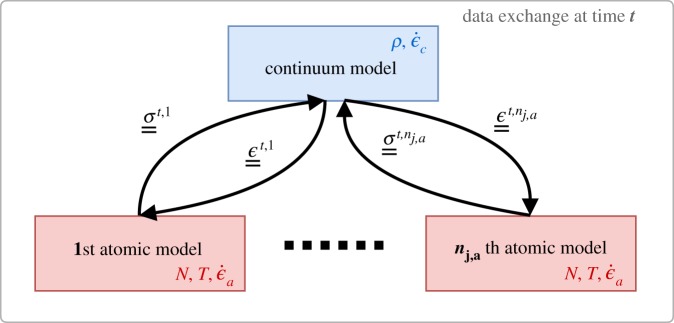
Data interactions between the upper (continuum) model and lower (atomistic) models simulated for each quadrature point *q* of the mesh of the continuum; the projected strain tensor ***ϵ*** is passed down, while the homogenized stress tensor ***σ*** is transferred up. The local density *ρ* and the strain rate ${\dot{\epsilon }}_{c}$ are constants of the continuum model, while the number of atoms *N*^*a*^, the temperature *T* and the strain rate ${\dot{\epsilon }}_{a}$ are constants of the atomistic model. (Online version in colour.)

Now to be more explicit, what we have so far referred to as the ‘mechanical state’ can be described via two standard variables of continuum mechanics: the infinitesimal strain ***ϵ*** and the Cauchy stress ***σ*** tensors. These variables constitute the sole information to be transferred between the two scales. In our implementation, ***ϵ*** is used as the input to the atomistic simulation, engendering a deformation to the new state (figure 4). The resulting change in pressure, and more specifically the virial form of ***σ*** (3.2) is then communicated back to the continuum model so that the balance of forces can be computed from the integral of ***σ*** over the whole continuum volume. Through this cyclic coupling, the time evolution of both the continuum and atomistic scale models is linked.

The virial form of ***σ*** (3.2):

3.2 $$\left\langle \sigma_{ij}\right\rangle =\frac{\left\langle \sum_{k=0}^{N^{a}}m_{k}^{a}v_{k,i}^{a}v_{k,j}^{a}\right\rangle }{V_{a}}+\frac{\left\langle \sum_{k=0}^{N^{a}}r_{k,i}^{a}f_{k,j}^{a}\right\rangle }{V_{a}}$$

is defined as the sum of the time-average of a kinetic term which is a function of the mass *m*^*a*^_*i*_ of the particle *i* and the velocity *v*^*a*^_*k*,*i*_ of the particle *i* in direction *k* and the time-average of a potential term which is a function of the position ***r***^*a*^_*i*_ and the exerted force ***f***^*a*^_*i*_ on the particle *i* of the *N*^*a*^ particles of the atomistic system of volume *V*_*a*_.

To simplify the exchange of information between the scales, we have made a few assumptions. As we apply a thermostat at the atomistic scale, the temperature is controlled and constant throughout the simulation; thus the effect of temperature gradients at the continuum scale is not included. In short, we assume that the atomistic description is always at local equilibrium. In the case of large inelastic deformations, this could become an issue, as heat is released due to friction or bond dissociation mechanisms. This effect could be mitigated by testing set-up characteristics, such as structures with limited bulk. Extending the current continuum model to account for complete thermodynamics could be a viable option because of the very small computational cost of macroscale versus micro-scale simulations. Also, there is no mass flow in our model, meaning the mass in each finite-element cell is conserved (and is constant at each *q*), hence the number of atoms is constant in each atomistic model. Indeed, the rate of deformation of the finite-element cells is assumed to be much slower than the time needed to relax the atomistic simulations. Consequently, we do not enforce that ${\dot{\epsilon }}_{a}$ be equal to ${\dot{\epsilon }}_{c}$, and allow both scales to evolve on their own time scales. In the case of a much greater ${\dot{\epsilon }}_{c}$, with the finite-element cell deforming faster than *τ*_*a*_, then this assumption would need to be revisited.

It is relatively trivial to communicate ***ϵ*** at a given quadrature point to the corresponding atomistic model via a boundary condition (***ϵ*** is an internal variable of the continuum model). However, the reverse coupling—transferring the resultant ***σ*** back up to the continuum model—is a more complicated task (and is described below). The stress sampling method employed is an average of the mechanical state over the whole atomistic system (see equation (3.2)). In turn, such a method is less sensitive to fluctuations and therefore converges rapidly. However, it enforces homogeneity on the sampled stress state. More sophisticated and tightly coupled techniques exist for bridging from discrete to continuum medium [40,41] which capture stress heterogeneity and avoid spurious stress reflection although they suffer from the requirement of much greater computational costs compared to HMM.

The desired deformation (corresponding to ***ϵ*** obtained from the upper model) is applied to the atomistic system via a gradual affine transformation of ***r***^*a*^_*i*_ (option ‘remap x’ in LAMMPS). This is performed such that the increment of strain applied during the evolution of the atomistic model matches the largest ${\dot{\epsilon }}_{a}$ possible while enabling the system to remain relaxed. That is to say, ${\dot{\epsilon }}_{a}$ is sufficiently small such that the deformation is applied over a period far longer than *τ*_*a*_. *τ*_*a*_ is expected to be orders of magnitude smaller than Δ*t*_*c*_, in order for the assumption of the separation of scales to be valid, therefore ${\dot{\epsilon }}_{a}$ is generally much larger than ${\dot{\epsilon }}_{c}$.

### (d) Sampling the stress tensor

The stress tensor ***σ*** at a given location in the continuum model is derived from the atomistic model. Unlike in the case of the strain tensor ***ϵ***, ***σ*** is not an internal variable of the atomistic model, and a homogenization procedure must therefore be performed on the virial (3.2). Owing to fluctuations in the motion of the atoms, this homogenization procedure must be performed not only across space, but also across time. ***σ*** is computed once the system has become stationary following its deformation. Fixed dimensions, number of atoms and temperature (i.e. the use of the NVT ensemble in LAMMPS) are applied throughout the homogenization procedure. Spatial homogenization is performed over the entire sample , assuming it is free of finite-size effects and simulated using periodic boundary conditions.

Time homogenization (or sampling), however, is a more arbitrary procedure. There exist many approaches in the literature on this subject [42,43]. Ideally, one would allow the atomistic system to evolve under the given constraints (NVT) for a sufficiently long time such that all the possible configurations are observed. To render this computationally tractable, we choose to perform an ensemble averaging of the ***σ*** over a shorter period of time [44,45]. Instead of running a single simulation per *q*, we instead run several replicas, and perform time averaging on each of them for a period proportional to the autocorrelation length of the system's pressure.

**Figure 5.**
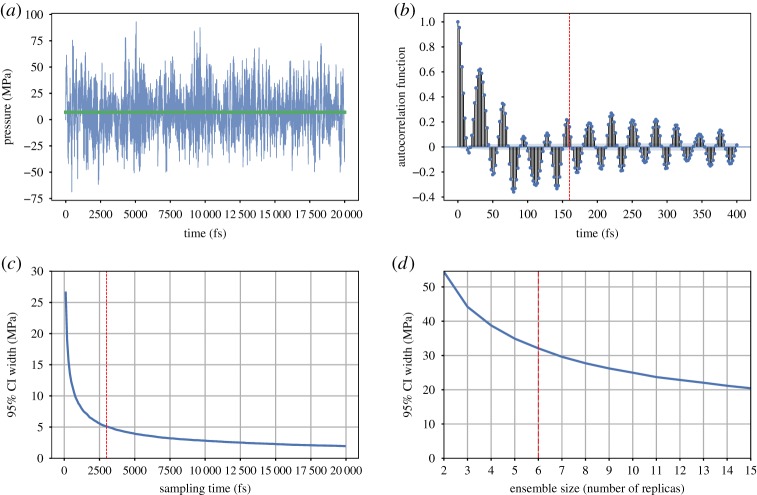
Stress sampling procedure parametrization; (*a*) fluctuation of the pressure of a single replica of the atomistic system at 10% uniaxial stretch over a period of 20 picoseconds, the average pressure is shown in green; (*b*) the autocorrelation function of the pressure time series shown in (*a*), the red dotted line showing the value of the autocorrelation time length; (*c*) the evolution of the confidence interval of the time-averaged pressure with respect to the duration of sampling, the red line showing the optimal duration sampling; (*d*) the evolution of the confidence interval of the ensemble averaged pressure over the optimal duration with respect to the number of replicas *n*_*r*_, the red line showing the optimal sampling ensemble size. (*a*) Pressure fluctuation, (*b*) pressure autocorrelation function, (*c*) time averaging length and (*d*) ensemble size. (Online version in colour.)

To completely define the homogenization procedure, we must now determine the appropriate number of replicas *n*_*r*_ to simulate and the duration (as a function of the autocorrelation length) over which to average. This is determined by observing the evolution of the uncertainty (95% confidence interval) on the homogenized pressure (i) in a single replica, to determine the optimal duration of sampling (figure 5*c*), and given this optimal duration (ii) in an ensemble of replicas, to determine the optimal *n*_*r*_ (figure 5*d*). We adapt these two parameters to reach a sufficient accuracy. The confidence interval appears to reduce as a power law of the sampling duration and *n*_*r*_. To select the optimal trade-off between accuracy and computational cost, we choose values corresponding to the point of maximum curvature of the power laws, beyond which we consider that the gain in accuracy is not worth the increase of cost of computation (in particular with regard to the *n*_*r*_).

**Figure 6.**
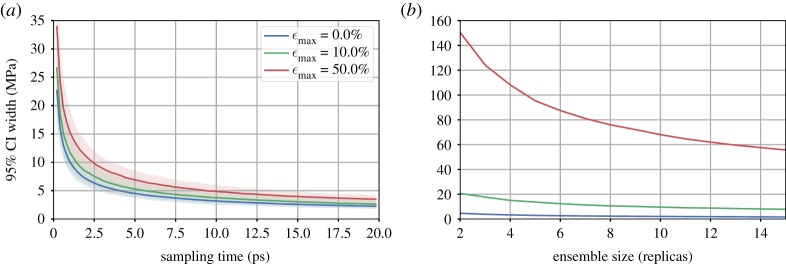
Influence of the mechanical state on the sampling duration (*a*) and ensemble size (*b*) for TGMDA epoxy; stretching the epoxy system reduces local geometrical constraints such as friction between the strands, in consequence longer sampling duration is needed and variations of pressure among replicas are much larger. (*a*) Sampling duration and (*b*) ensemble size.

The homogenization procedure is performed for different mechanical states (figure 6). Axial deformations of 10% and 50% described above are applied to the tetra-functional epoxy system and compared with the same system at equilibrium (0% deformation). As the system is stretched, the duration of sampling can be slightly longer. Up to 50%, the deformation reorganizes the polymer structure in a looser way, growing voids and reducing friction, with geometrical constraints becoming weaker. Also, the number of replicas *n*_*r*_ needed to reach a given confidence interval increases drastically. At high strains, mechanical instabilities can cause large differences in the response of different replicas to an identical mechanical state. To ensure a certain accuracy of the predicted ***σ***, it may become necessary to increase the *n*_*r*_ when simulating at extreme mechanical loads.

### (e) Heterogeneous multiscale method-oriented single-scale model considerations

Despite most of the subtleties of the implementation of HMM lying in the homogenization procedure, it is worth commenting on aspects of the single-scale models which can impact the efficiency of the global workflow.

#### (i) Atomic interaction potential parametrization

On the atomistic level, the choice of the force field has an important impact on the computational cost of the overall model, and is selected with consideration of the quantities of interest at the continuum level. The main distinction we make here is that between reactive and non-reactive force fields. Depending on the material and the mechanisms we need to capture at the continuum level, one may choose to employ a reactive force field. Reactive force fields, such as ReaxFF [46], EAM [47] and AIREBO [48], enable the topography of covalent bonds to evolve throughout a single simulation of the atomistic model. However, this enhancement comes at the price of important overheads—the authors of the ReaxFF claim an increase of 10–50 times of the computational costs [49]. In a comparison of two simulations of a uniaxially stretched epoxy resin system of approximately 40 000 atoms with a Nosé–Hoover thermostat, one with the non-reactive OPLS force field and the other with ReaxFF, we observed on average a 10-fold increase in the time to compute a single time-step with ReaxFF. Furthermore, the typical OPLS time-step of 1–2 fs needed to be reduced to 0.5 fs to ensure dynamic stability of the simulation with ReaxFF, leading to a 20–40-fold slow down overall. Such significant overheads must be taken into account when designing these multiscale virtual experiments.

In the case of thermoset polymers, it is not possible to capture the mechanisms leading to failure of the material without modelling bond breakage, as conformational changes are constrained by the cross-linking. Reactive force fields are therefore necessary and ReaxFF is employed in §4. Conversely, in the case of thermoplastic polymers, failure occurs via disentanglement and sliding of chains. Rupture of bonds may not be the determining factor in such cases.

#### (ii) Continuum model solution algorithm

At the level of the continuum model, the time and spatial discretization will naturally influence the computational expense, as it impacts directly *n*^*t*^_md_. However, the choice of solution algorithm also plays a determining, though more intricate, role.

To predict the displacements of the nodes of the mesh of the continuum model ***u*** (thus determining the local strains to be transferred to the atomistic models), the evolution of the continuum system may be handled either quasi-statically (3.3):

3.3 −∇σ(u)=ρf

or dynamically (3.4):

3.4 $$\nabla \sigma \left(u\right)+\rho f=\rho \frac{\partial^{2}u}{\partial t^{2}}.$$

These two main options have different requirements with respect to the atomistic system.

Solving for static equilibrium involves inverting the relation between external and internal forces of the continuum system. Internal forces can be expressed as a linear function of ***u***, and a global stiffness matrix ***K*** (3.5):

3.5 $$u^{t+1}=K^{-1}\frac{F_{e}^{t+1}-F_{e}^{t}}{\Delta t_{c}}$$

with ***F***_*e*_ the external nodal forces applied on the discretized continuum.

**Figure 7.**
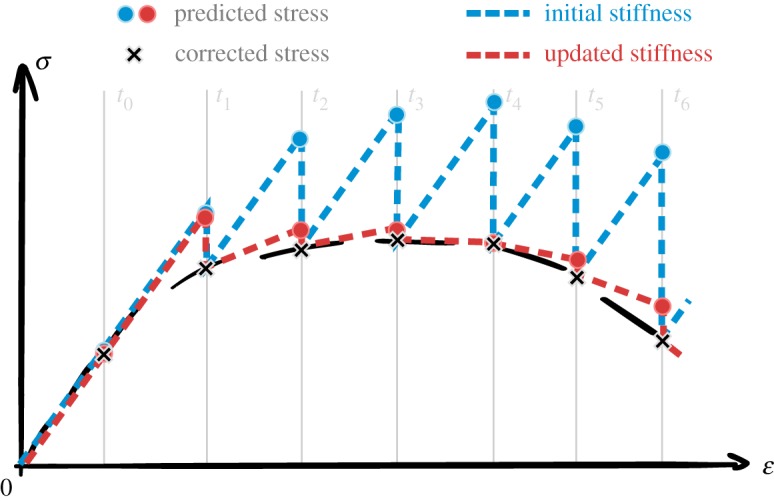
Solving quasi-static equilibrium of continuum mechanics implies evolving the continuum system using the stiffness matrix ***K*** of the associated linearized system of equations; which relies on the initial estimation of $\tilde{C}$ at equilibrium (blue) or on updated estimations all along the deformation path using the atomistic system (red); reducing the frequency update leads to larger error on the estimation of ***u*** in between prediction-correction steps. (Online version in colour.)

The global stiffness matrix depends directly on $\tilde{C}$ of the material in its current state. Unlike the mass properties, $\tilde{C}$ varies as the atomistic system evolves, and inelastic mechanisms occur such as covalent bond breaking or chain disentanglement. Therefore, the stiffness matrix needs to be recomputed frequently to avoid accumulation of error during the prediction of displacements (figure 7), while the mass matrix ***M*** is only assembled once, initially. As discussed in section (3.e.i), the evaluation of $\tilde{C}$ carries a significant cost, and reducing the frequency of their calculation is essential to the feasibility of the overall simulation. Another potential speed-up is to only compute an approximation of $\tilde{C}$ derived from the interatomic potential expression omitting the stress fluctuation term [27].

Solving for dynamic equilibrium relies on methods such as the Euler forward or backward schemes. The force balance is expressed as a function of the accelerations ***a***, and this relation is then inverted to obtain accelerations (3.6):

3.6 $$a^{t+1}=M^{-1}\frac{F_{e}^{t+1}-F_{e}^{t}}{\Delta t_{c}}.$$

In turn, the node velocities ***v*** and ***u*** are successively derived. The solution algorithm only requires knowledge of the mass properties of the continuum system, which have the benefit of being stationary (due to the zero mass flow condition). The mass matrix of the system, inverted to obtain the nodal accelerations, is built only once during the initialization stages of the simulation, and using the masses of the atomistic systems.

Consequently, as part of an HMM, solving dynamic equilibrium is rather more convenient, although several drawbacks persist. For example, the time and space discretization constraints necessary to ensure stability of integration may, in turn, increase the sum of *n*^*t*^_md_. Classically, stability is ensured by enforcing the Courant–Friedrichs–Lewy condition [50] (3.7):

3.7 $$\frac{\Delta x_{c}}{\Delta t_{c}}<∥\underline{v}{∥}_{l_{2}.}$$

## 4. Prediction of fracture toughness informed by chemical composition

**Figure 8.**
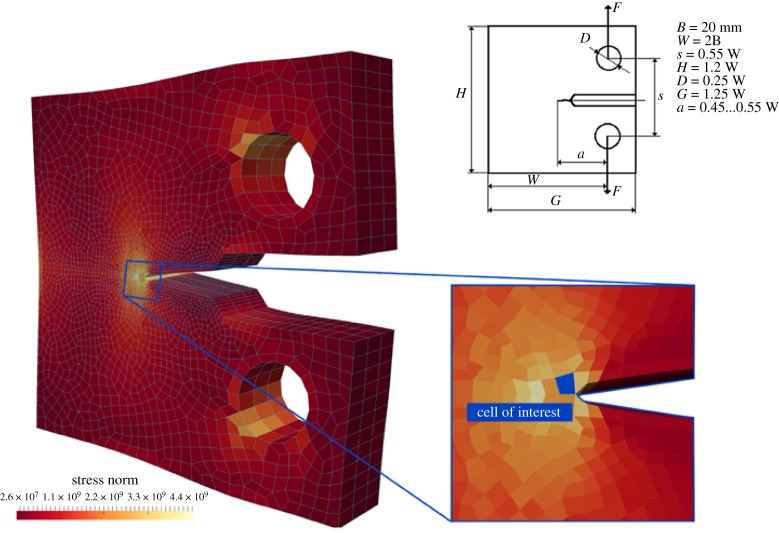
The standard ASTM ‘compact-tension’ test set-up dimensions (top, right); vertical eccentric pulling of the holes generates a tensile mechanical state localized in the notch tip as illustrated by the stress field (∥***σ***∥); the mesh of the continuum model (in blue, left) is refined in the vicinity of the notch tip; the blue-filled cell (bottom, right) is used for the detailed mechanical analysis in figure 9. (Online version in colour.)

Fracture toughness is used here as a classic example of a mechanical property that is of utmost importance to engineers when looking for new structural materials. The evaluation of fracture toughness of a new material is typically performed experimentally by means of what is called a ‘compact-tension’ test following ASTM International Standards [51] (ASTM/E1820). The geometry of the test is specifically designed to trigger the appearance of a single crack in the structure. The dimensions of the compact-tension test specimen are typically of the order of a few centimetres, while the load is applied over a few seconds (figure 8).

**Figure 9.**
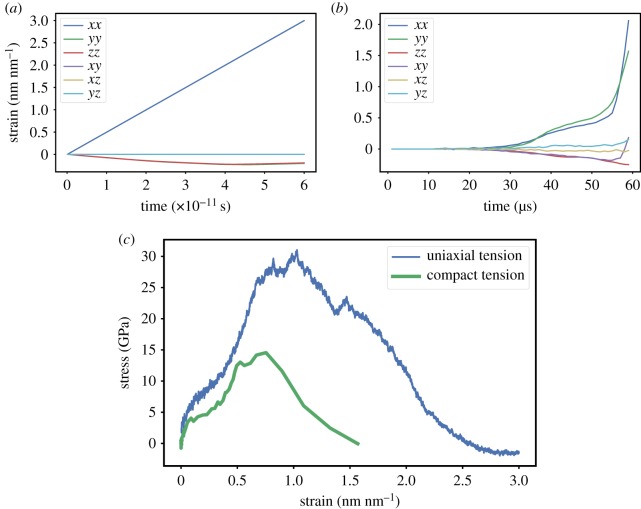
The set-up of the tension test has significant influence on the local mechanical state at the atomistic scale; in a uniaxial tension test (*a*) the continuum strain tensor is purely diagonal; while in a compact tension test (*b*) the continuum strain tensor is rather bi-dimensional comprising tension in two directions and shearing in the same plane; in turn, the constitutive behaviour (*c*) in the direction of failure (normal to the crack plane) displays different yield, strength and energy dissipation; the epoxy resin appears much tougher when loaded in a uniaxial tension test. (*a*) Uniaxial tension strain evolution, (*b*) compact tension strain evolution, (*c*) stress–strain relation.

When considering a chemically specific atomistic description of the material to be characterized, in this case an epoxy resin, the testing set-up to determine the fracture toughness must be simplified as the time and space dimensions of the ASTM test cannot be computed at atomic resolution. Fortunately, fracture toughness is defined as the energy required to split a piece of material in two. In all-atom MD studies of fracture properties, the ASTM experimental set-up is often simplified to a uniaxial tension test performed on a cubic specimen a few nanometres long, over a few nanoseconds [28–31]. Leaving aside the limitations of performing such a test over very short time scales, and therefore missing time-dependent mechanisms characteristic at the microsecond scale or above, we focus here on the validity of the simplification of the ASTM compact-tension test as a uniaxial tension test. We compare the local response of the material characterized with the two tension test set-ups. For the compact tension set-up, we focus on a cell located at the notch tip (figure 8). The TGMDA epoxy is used as the material for comparison. In both set-ups, ${\dot{\epsilon }}_{a}$ is a chosen set according to the convergence value in figure 5, namely 0.0001 fs^−1^. Similarly, the number of replicas and the stress sampling time are set as prescribed, respectively, in figures 5*d* and 5*c*, that is six replicas and 3 ps.

For the simple case of a single-scale atomistic MD simulation, the only non-negligible strain component lies in the direction of loading (figure 9*a*), and residual strains are observed laterally. However, in the HMM case, the deformation of the atomistic model is continuously informed by the evolution of the macroscopic continuum model, i.e. the deformation at the notch tip of the compact tension test (figure 8). A different, complex mechanical state is therefore observed (figure 9*b*) involving bi-axial tension and shearing. These diverging applied strains lead to different apparent responses of the material in the orthogonal direction to the failure plane. The evolutions of the stress–strain relation for the two set-ups are compared in figure 9*c*. Both set-ups exhibit the same qualitative features: a short elastic regime followed by yielding, hardening and brittle failure at very large strains. However, quantitatively these inelastic events occur within a shorter strain range in the case of the compact tension test. Correspondingly, the HMM simulation of the compact tension test predicts the apparent fracture energy to be significantly lower.

**Figure 10.**
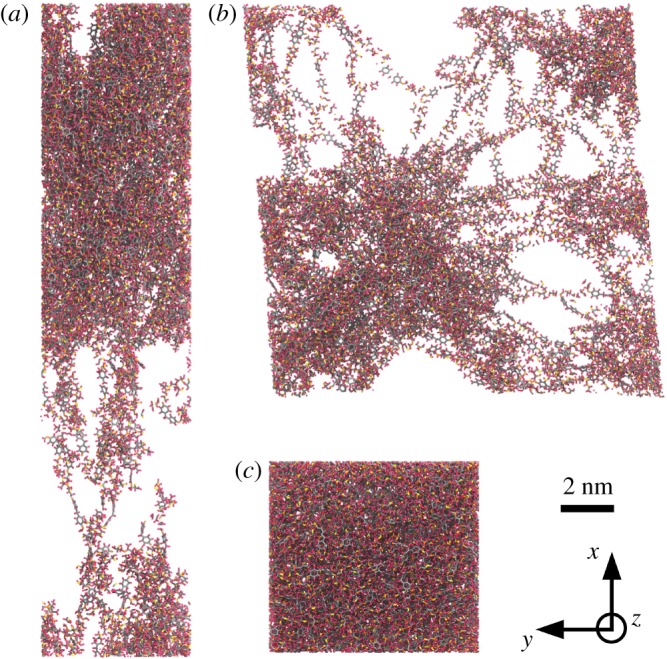
Conformations of the epoxy at the onset of fracture under uniaxial tension boundary conditions (*a*) and under compact tension boundary conditions applied using the HMM (*b*), and at equilibrium (*c*) for reference. The deformations applied in (*a*) and (*b*) correspond to the strain tensors shown in figure 9. These configurations are obtained from the same replica of the atomistic system which contains 38 611 atoms. The carbon atoms appear in grey, the nitrogen atoms in yellow, the oxygen atoms in red and the hydrogen atoms in pink. The two tension set-up displays highly contrasted conformational changes in the final state of the atomistic system, in particular void growth is more dispersed with the compact tension set-up, potentially exhibiting two orthogonal crack planes.

The divergence of strains applied in the two set-ups has additional implications when observing the conformations of the resins at the onset of fracture, as shown in figure 10. The epoxy network under uniaxial tension exhibits localized void growth leading to a single continuous crack, orthogonal to the tension direction (figure 10*a*). Conversely, under the deformation conditions at the notch tip of the compact tension set-up, the observed void growth in the epoxy network is more dispersed (figure 10*b*). The resulting fracture pattern is less clearly defined. One can distinguish two competing cracks, orthogonal to the two tension directions. The HMM captures different local failure mechanisms, which are responsible for the differences in stress–strain behaviour observed above. Appropriately emulating the set-up of the standard testing procedure is therefore indispensible for accurate macroscopic property prediction, and would be difficult to reproduce efficiently without a multiscale approach.

Given the importance of accurately determining the local strain evolution through consideration of the broader balance of forces further afield, one might consider integrating the mechanics of the compact tension test using a hierarchical multiscale approach to avoid numerous simulations of the atomistic model. As such, one would miss the opportunity to observe the local mechanisms explaining the response of the material (void growth, in the present case). Additionally, one would require a mechanically accurate and chemically specific set of constitutive equations relating ***σ*** and ***ϵ***. Constitutive models, being purely phenomenological and lacking numerical robustness, must often be formulated subject to supplementary constraints. An efficient and physically justified constraint can be to enforce that the constitutive equation be thermodynamically admissible [52]. This requires ***σ*** to derive from a convex potential and the dissipated energy to increase monotonically. The need for robustness has significantly limited the complexity of such constitutive models, leaving them only able to reproduce relatively simple mechanisms such as isotropic damage (4.1) [53] or plasticity [54,55]:

4.1 $$\begin{array}{r} \sigma_{ij}=(1-D)E\epsilon_{ij}\\ \mathrm{and}\;D=f\left({\left\langle \epsilon \right\rangle }_{+}\right) \end{array}\}$$

with *D* the scalar damage variable, varying from 0 to 1 and reproducing the level of degradation of the material, and *E* the Young modulus of the pristine material; 〈.〉_+_ is the positive part of a given matrix, computed as the positive part of the eigenvalues.

Figure 9*b* illustrates how complex the actual constitutive behaviour can be, and how simple isotropic models would be unsatisfactory. Indeed, constitutive behaviour informed by processes at the atomistic scale would comprise anisotropic yield, hardening and softening. Furthermore, chemical specificity is poorly captured in the constitutive equation due to the difficulty of estimating parameters that often lack direct physical meaning (e.g. the parameters in damage models, which are standard and robust models designed to reproduce fracture in a continuum model).

## 5. Computational workflow

Keeping track of inelastic mechanisms requires storage of the (history-dependent) current state of the material at each spatial location. As constitutive behaviour is handled via atomistic simulations, the continuum level need only know the current strain and stress tensors at each *q*. Conversely, at the atomistic level, the requirements imposed by inelastic mechanics can be more cumbersome. History dependency implies that the atomistic system must be brought to the desired mechanical state, with the exact ${\dot{\epsilon }}_{a}$ and following the exact straining trajectory, otherwise different current mechanical state could result. Evolving the atomistic system with a non-arbitrary ${\dot{\epsilon }}_{a}$ is expensive, and it rapidly becomes impossible to reach the current mechanical state starting from the initial one. Consequently, the most recent state of the atomistic system computed for each integration point of the continuum model must be stored. This amounts to a significant trade-off between memory and computational cost.

The resulting HMM workflow is distributed across many cores and can display significant load-imbalance. The workflow is divided into a single continuum model and multiple atomistic models. At any given time-step, evolving the continuum model is generally far less expensive than evolving one of the many atomistic models (being of the order of 0.1% to 1% of the computational effort). The continuum and atomistic models are evolved alternatively, but the atomistic models themselves are run in parallel, drastically improving the scalability of the method. Nonetheless, there is a highly variable difference in resource requirements between continuum and atomistic simulation phases. This might justify performing the simulation of the continuum model on a separate smaller resource. In turn, we would avoid idleness of a large part of the total allocation when the continuum model is evolved. *n*^*t*^_md_ can be assumed to be constant, but with individual execution times determined by the norm of the applied strain on the atomistic system (which may be zero). As such, efficient scheduling of the many atomistic simulations is non-trivial, and can involve allocating different numbers of cores to different simulations in an attempt to achieve (approximately) equal runtime. This problem could be tackled with the help of resource optimization libraries, which efficiently schedule each atomistic model simulation by taking into consideration queueing time, execution time and energy consumption across distributed resources [22].

## 6. Conclusion

We have described a HMM implementation suited for the study of nonlinear irreversible macroscopic mechanisms and its rigorous testing and application to polymer thermosets. We have discussed in detail the resulting computational workflow and associated numerical tools to solve multiscale problems encompassing atomistic and continuum descriptions of the material. We described an implementation to determine the sampling requirements for homogenization of the material behaviour at the atomistic level and the transfer of the associated information to the continuum level.

Via an application to the simulation of the measurement of the fracture properties of an epoxy resin, we illustrate the benefits of the HMM relative to a single-scale atomistic approach. Unlike single-scale or even hierarchical multiscale models, our multiscale approach efficiently handles the complexity induced by the local mechanical behaviour of the material, while integrating the influence of the boundary conditions imposed by the testing set-up at the engineering scale. We have shown a significant quantitative difference in stress–strain response and fracture toughness predicted by a purely single-scale (MD) approach and that predicted by our HMM implementation. Simulated degradation mechanisms, such as void growth, also differ substantially. This demonstrates the necessity of using a concurrent multiscale approach for accurate and chemically specific prediction of mechanical properties at the macroscale.

## Data accessibility

The HMM workflow developed in the present paper is available on GitHub: https://github.com/mvassaux/DeaLAMMPS.

## Glossary

*D*_*a*_: spatial length of an atomistic system.
*D*: scalar damage variable of an isotropic damage constitutive law.
*E*: Young modulus as defined by Hooke's law.
*N*^*a*^: number of particles in the atomistic model.
*T*_*a*_: time duration of an atomistic model simulation.
*T*: ensemble temperature of the atomistic system.
*U*: ensemble potential energy of the atomistic system.
*V*_*a*_: volume of an atomistic system.
Δ*t*_*c*_: time duration of a time-step of continuum model incremental solution algorithm.
Δ*x*_*c*_: spatial length of a finite-element cell of the mesh of the continuum model.
***F***_*e*_: external forces of the nodes of the mesh of the continuum model.
***R***(*t*): a stationary Gaussian process with a zero time average introduced by Langevin dynamics.
***a***: accelerations of the nodes of the mesh of the continuum model.
***f***^*a*^_*i*_: force vector of the particles in the atomistic model.
***f***: local volumic forces applied to the continuum system.
***r***^*a*^_*i*_: displacement vector of the particles in the atomistic model.
***u***: displacements of the nodes of the mesh of the continuum model.
***v***: velocities of the nodes of the mesh of the continuum model.
***x***: positions of the nodes of the mesh of the continuum model.
***ϵ***: infinitesimal strain tensor.
***σ***: Cauchy stress tensor.
${\dot{\epsilon }}_{a}$: norm of the strain rate tensor of the atomistic system.
${\dot{\epsilon }}_{c}$: norm of the strain rate tensor of the continuum system.
*γ*: damping parameter introduced by Langevin dynamics.
***K***: global stiffness of the finite-element mesh of the continuum model.
***M***: global mass of the finite-element mesh of the continuum model.
*ρ*: local density of the continuum system.
*τ*_*a*_: characteristic time of the atomistic system.
$\tilde{C}$: tangent (or instantaneous) fourth-order stiffness tensor.
*gt*: scale separation computational gain in time.
*gx*: scale separation computational gain in space.
*k*_*B*_: Boltzmann constant.
*l*_*a*_: characteristic length of the atomistic system.
*m*^*a*^_*i*_: masses of the particles in the atomistic model.
*n*_*r*_: number of replicas of the atomistic system.
*n*^*t*^_*md*_: number of atomistic simulations at a given time-step *t*.
*q*: quadrature/integration points of the mesh of the continuum model.
*v*^*a*^_*k*,*i*_: velocities of the particles in the atomistic model.
FEM: finite-element method.
HMM: heterogeneous multiscale method.
MD: molecular dynamics.
TGMDA: tetraglycidyl methylene dianiline.
